# Effect of green tea dust as a dietary additive and anthelminthic on performance, digestibility, and fecal egg counts in Priangan ewe lambs infected with Strongyles worms

**DOI:** 10.1016/j.vas.2024.100395

**Published:** 2024-09-05

**Authors:** Diky. Ramdani, Aldyansah Putra. Utama, Ririn Siti. Rahmatillah, Juju. Julaeha, Novi. Mayasari, Ken Ratu Gharizah. Alhuur, Nanik. Hidayatik, Anuraga. Jayanegara

**Affiliations:** a Department of Animal Production, Faculty of Animal Husbandry, Universitas Padjadjaran, Jatinangor Campus, Sumedang, 45363, Indonesia; b Department of Animal Nutrition and Feed Technology, Faculty of Animal Husbandry, Universitas Padjadjaran, Jatinangor Campus, Sumedang, 45363, Indonesia; c Department of Animal Husbandry, Polytechnic of Agriculture Development (POLBANGTAN), Bogor, 16119, Indonesia; d Department of Veterinary Science, Faculty of Veterinary Medicine, Universitas Airlangga, Surabaya, 60132, Indonesia; e Department of Animal Nutrition and Feed Technology, Faculty of Animal Husbandry, IPB University, Bogor, 16680, Indonesia

**Keywords:** Anthelmintic, Digestibility, Green tea dust, Local lambs, Performance

## Abstract

Despite green tea dust (GTD) is a by-product of green tea fabrication, it has the potential as natural additive and anthelmintic in ruminant diets due to its high protein and bioactive contents. This study aimed to compare the effect of 3 different doses [0% (control), 0.75% (GTD-0.75), and 1.5% (GTD-1.5)] of GTD supplementation on dry matter intake (DMI, g/kg DM), average daily gain (ADG, g/kg DM), nutrient digestibility (%), and fecal egg counts (FEC, eggs/g feces) of local ewe lambs infected with *Strongyles* worms during 84 days feeding trial using a completely randomized design and six replicates (*n* = 6). The results showed that all lamb groups decreased body weight; however, GTD-1.5 supplemented lambs tended (*P* = 0.061) to have better ADG at the end of the trial compared to the other groups. In addition, GTD-1.5 supplemented lambs had lower (*P* < 0.05) FEC at day 42 and day 56 than those the control lambs. About 1.5% GTD supplementation in a diet has the potential as dietary additive and anthelmintic to maintain the productivity, health, and welfare in rearing lambs.

## Highlights

• Green tea dust is rich in protein and phenolic tannins.
• Dietary green tea dust helps *Strongyles* worms-infected lambs to improve their body weights and to reduce the nematode infection significantly.
• A proper dose of green tea dust inclusion in a diet is suggested to maintain the productivity, health, and welfare statuses of the rearing lambs.
• Green tea dust is a by-product in green tea fabrication and it is likely an affordable natural feed additive and anthelmintic for ruminants in high tea-producing countries.

## 1 Introduction

In many developing countries, rearing local sheep is one of important activities among farmers, especially in the rural areas. Local sheep are more preferable to be produced because they have good environmental adaptability and reproductive performances. In Indonesia, the sheep population has continued to increase from 2016 (15,716,667 heads) to 2019 (17,833,732 heads) (Central Statistical Bureau, 2020). Increased sheep population has been followed by an increase in sheep market demand, not only for daily lamb consumptions, but also several Islamic festivals such as Qurban and Aqiqah. The typical ruminant diets in most South East Asia (SEA) countries are formulated from low quality and fibrous feed ingredients such as paddy straw (Ramdani, Budinuryanto & Mayasari, 2020). A concentrate supplementation is greatly required to improve the quality of paddy straw-based diet for sheep. In fact, the fibre fractions of typical concentrates in SEA countries such as Indonesia are high since they are commonly produced from a mixture of fibrous agricultural by-products. Thus, a relevant dietary additive is considerably required to be added in a concentrate diet to improve its quality to enhance sheep productivity, health, and welfare.

Nematodiasis is a disease that often occurs in a tropical country such as Indonesia and is supposed to be a major obstacle among farmers to increase the productivity of their sheep and goats (Ekawasti et al., 2017; Julaeha, Ramdani & Setyowati, 2021). Nematodiasis is one of parasitic infected disease and must be controlled intensively (Purwaningsih, Kusumastuti, Sumiarto & Sumiarto, 2017) as infected sheep will have a disrupted body growth leading to gradual weight losses or even death in a worse case. Anugerah et al. (2023) suggests that nematode infections is not only affecting the performance and health, but also the welfare statuses of the livestock.

Many studies have reported the efficacy of plant-based medicines as dietary additive and anthelmintic in various ruminant diets (Ramdani, Yuniarti, Jayanegara & Chaudhry, 2023). One of potential plant bioactive compounds is tea polyphenols. Epigallocatechin gallate and the other catechin derivatives are the main bioactive constituents in tea polyphenols (Ramdani, Jayanegara & Chaudhry, 2022). Ramdani et al. (2020) found that green tea dust (GTD) was rich in total phenols and tannins and its supplementation into a diet resulted in increased performance of sheep without affecting nutrients digestibility. Julaeha et al. (2021) reported that *Jatropha multifida* was rich in total phenols and tannins and its dietary inclusion in a diet could increase the performance and reduce *Trichostrongylus* sp. Infection in growing lambs. Green tea dust is a co-product during green tea fabrication and its price is much lower than the original green tea. However, study on the potential use of GTD as an anthelmintic in a sheep diet has not been done yet. Thus, this study aimed to test the hypothesis that GTD supplementation can play multiple roles, not only as additive but also anthelmintic in the diet of local sheep infected with *Strongyles* worms.

## 2 Material and methods

### 2.1 Animals and handling

The research objects were 18 rearing ewe lambs (of Priangan breed, decree of Indonesian Agricultural Minister No. 300/Kpts/SR.120/5, 2017) at about 10 months of age with an average initial body weight of 16.1 kg and a coefficient of variation 14.8%. The experiment involved 3 treatment groups, each consisting of 6 ewe lambs (*n* = 6). The use of animals in this experiment has been approved by Universitas Padjadjaran Research Ethics Commission number: 307/UN6.KEP/EC/2021.

The lambs were adapted for 21 days to the experimental basal diet (300 g concentrate and 1 kg paddy straw silage per head per day) and the individual pen (1.1 m long x 0.7 m width x 1 m height) equipped with a feed trough and a water bucket. During adaptation, each lamb obtained an intramuscular injection with 1 ml per head of B complex vitamins (Injekvit B-Plex, PT. Medion Farma Jaya, Indonesia) and a subcutaneous injection with 0.5 ml of 1% Ivermectin (Ivomec, Merial Inc., imported by PT. Romindo Primavetcom, Indonesia). Ivermectin deworming treatment was applied to clean the worm infections in each lamb. After adaptation to the experimental diets and environment, each lamb was weighed before morning feeding using a digital weighing scale with 0.1 kg accuracy (Avery Weigh-Tronix, type E1005) to measure an initial body weight. After that, each lamb was repeatedly weighed before morning feeding at 14, 28, 42, 56, 70, and 84 days of the feeding trial to measure repeated body weights (kg) and average daily gains (ADG, g/head/day).

### 2.2 Experimental diets

Each experimental diet was offered 2 times: morning at 07.00 and evening at 16.00 (Jakarta time). Each GTD treatment was added and mixed thoroughly into the concentrate before it was offered to the ewe lambs. The concentrate (300 g/head/day) was offered in the morning, ensuring an even distribution of the supplement across all feeds provided. Paddy straw silage (1000 g/head/day) was offered after concentrate feeding and continued in the evening. Provision of clean drinking water was provided ad libitum. Each feed refusal was weighed by a digital scale (1 g accuracy) in the next morning day to calculate dry matter intake (DMI, g/head/day). Feed conversion ratio (FCR) was calculated by dividing DMI with ADG.

The diet ingredients consisted of paddy straw silage, concentrate, and GTD. The details of the experimental diet composition (DM basis) can be seen as follow:

Control:Paddystrawsilage(60%)+concentrate(40%)

GTD−0.75:Control+GTD(0.75%)

GTD−1.5:Control+GTD(1.5%).

Paddy straw was collected as waste products from several local rice farms located in Tarogong Kaler subdistrict, Garut regency, West Java. Paddy straw silage was prepared with the help of a probiotic starter. A total of 1 cc of Probiotic EM4 (Effective Micro Organism 4, PT. Songgolangit Persada, Indonesia) was mixed with 1 liter molasses and diluted in water at a ratio of 1:9. A total of 4 liters of probiotic mixture (4%) was sprayed to about 100 kg of the straw and stored in an aerobic and compact environment in the blue plastic barrel with steel lid fastener. After that, fermentation was carried out for a minimum of 14 days. Concentrate was formulated with locally available ingredients such as rice bran (19%), coffee husk (15%), palm kernel meal (15%), copra meal (15%), cassava meal (10%), local soybean meal (15%), corn germ feed (5%), dried distiller grains (5%), salt (0.40%), lime (0.50%), and Premix (0.10%, Kalvimix RX, Kalbe Animal Health) while GTD (Code: Dust 60, Grade: 3) was obtained from PT. Kabepe Chakra, Indonesia. Chemical compositions each of feed ingredients were shown in Table 1.

**Table 1:** Mean (*n* = 2) nutrient contents of the feed ingredients.

Contents	Paddy straw silage	Concentrate	GTD
DM (%)	26.4	87.5	96.3
Ash (%)	21.4	10.9	6.22
CP (%)	6.88	12.4	23.8
NDFom (%)	56.2	44.5	28.5
ADFom (%)	38.2	28.0	20.9
EE (%)	2.29	12.1	4.49
ME (MJ/Kg)	3.84	6.70	4.85
Total Phenols (%)	n.a	5.86	24.9
Total Tannins (%)	4.80	1.75	21.5

DM: Dry Matter; CP: Crude Protein; NDFom: Neutral Detergent Fiber Organic Matter; ADFom: Acid Detergen Fiber Organic Matter; EE: Ether Extract; ME: Metabolisable Energy; n.a: not available.

### 2.3 Infective larvae (L3)

Infective larvae (L3) preparation was done using a similar procedure to Julaeha et al. (2021). Briefly, each lamb was at worms-free condition after being treated with Ivermectin. After that, the infective larvae (L3) of Strongyles (Haemonchus sp., Cooperia sp., and Trichostrongylus sp.) were infested orally as many as 5000 larvae per sheep using a drenching gun. Larvae infestation was applied 3 times and the examination of fecal egg counts (FEC, eggs/g feces) was carried out in the 3rd week. The initial FEC (Day 0) was 808.9 ± 211.5 eggs/g feces.

### 2.4 Fecal egg count (FEC, eggs/g feces)

Adequate feces were collected using grab sampling method from the rectum of each lamb at 0 (day 0, three weeks after oral larvae infestation), 14 (day 14), 28 (day 28), 42 (day 42), 56 (day 56), and 72 (day 72) days of the feeding trial. The examination of FEC was done using the floating and Whitlock counting slide methods (Nematollahi, Moghaddam & Nyiazpour, 2007). Briefly, about 3 g of each feces sample was weighed and placed in a beaker glass, to which 17 mL of water was added and stirred. Subsequently, approximately 40 mL of saturated salt solution was added to the beaker glass and mixed until being homogeneous. About 0.5 mL of the mixed solution was further collected using a pipette and put into a Whitlock counting slide and the worm eggs were identified and counted under a microscope with 40 × magnification. The FEC result as eggs/g feces was calculated by multiplying the number of discovered worm eggs with 40 (Ekawasti et al., 2017; Julaeha et al., 2021). The identifications of eggs and worm larvae types followed the Royal Veterinary College method (Van Wyk & Mayhew, 2013; Zajac & Conboy, 2012).

### 2.5 Nutrient digestibility

Dry matter (DMD, %), organic matter (OMD, %), and protein (%) digestibility trial were done using a 7 days total feces collection method as described in Ramdani et al. (2020) at the end of 84 days feeding trial.

### 2.6 Chemical analysis

Either paddy straw silage or concentrate in several blue plastic barrels or sacks, respectively, was randomly sampled in three or five different parts and pooled. Each sample was dried in an oven drying at 60 °C for about 48 h. Each dried sample was then ground to pass through a 1-mm sieve in a sample disc mill before being subjected to various nutrient analyses using standard protocols of the Association of Official Analytical Collaboration (AOAC, 2005) to determine crude protein (CP, AOAC 990.03), ash (AOAC 942.05), and ether extract (EE, AOAC 920.39). The neutral detergent fibre (NDFom) contents were analyzed using the procedure of Van Soest, Robertson and Lewis (1991) without using amylase and decalin while acid detergent fibre (ADFom) was determined using the method of Van Soest (1973). Metabolisable energy (ME) was analyzed using the method of Menke and Steingass (1988) as described in Ramdani et al. (2022). Total phenols (TP) and total tannins (TT) were analyzed using the Folin–Ciocalteu method (Makkar, 2003; Ramdani et al., 2020). All nutrient contents were express as percentage DM except DM and ME, which were expressed as a percentage of fresh samples and MJ/ kg, respectively.

### 2.7 Statistical analysis

Each chemical content of the feed materials was calculated as an average from duplicate analysis (*n* = 2). A completely randomized design was used to compare the effect of 3 different doses (Control, GTD-0.75, and GTD-1.5) of GTD in a rearing diet on the performance, nutrient digestibility, and FEC in *Strongyles* infected local ewes during the trial using 6 replicates (*n* = 6). The data were statistically analyzed using one-way ANOVA in MINITAB 16 statistical software, in which Tukey's test was applied to compare means. Statistical significance was set at *P* ≤ 0.05. The residual data were analyzed for normality by passing the Anderson–Darling normality test at *P* > 0.05.

## 3 Results

Adding GTD into a rearing diet did not affect (*P* > 0.05) ADG, DMI, and FCR of rearing lambs infected with *Strongyles* worms. However, GTD-1.5 treatment tended (*P* = 0.061) to increase ADG of the lambs at the end of 84 days feeding trial in comparison with other treatments (Table 2).

**Table 2:** Effect of different green tea dust (GTD) supplementations (*n* = 6) on body weight (BW, kg), average daily gain (ADG, g/head/day), dry matter intake (DMI, (g/head/day), and feed conversion ratio (FCR) during 84 days feeding trial.

Measurement	Control	GTD-0.75	GTD-1.5	SEM	*P-value*
Initial BW (kg)	17.3	15.0	15.9	0.95	0.275
Final BW (kg)	15.8	13.8	15.7	1.01	0.309
ADG (g/head/day)					
Day-14	21.2	5.34	8.85	11.1	0.579
Day-28	0.04	−18.7	−8.22	0.98	0.290
Day-42	−9.36	−16.3	−4.80	6.75	0.496
Day-56	−18.7	−25.2	−2.11	7.94	0.140
Day-70	−12.5	−16.8	−2.14	7.84	0.418
Day-84	−17.2	−14.6	−2.16	4.35	0.061
DMI (g/head/day)					
Day-14	343	338	337	4.59	0.574
Day-28	345	339	338	4.31	0.522
Day-42	345	338	337	10.1	0.296
Day-56	342	335	333	3.90	0.254
Day-70	338	331	329	4.09	0.324
Day-84	489	450	474	17.1	0.300
FCR					
Day-14	−67.4	9.3	29.2	114	0.821
Day-28	−106	−12.6	−5.0	77.5	0.604
Day-42	−115	34.8	−6.4	68.3	0.306
Day-56	−67.8	−27.3	−9.66	26.4	0.308
Day-70	207	−154	−11.4	139	0.215
Day-84	−66.2	−45.5	−7.53	36.4	0.528

The same letters in the same column indicate a non-significant different at *P* > 0.05.

In addition, GTD supplementation showed no impact (*P* > 0.05) on DMD, OMD, and protein digestibility of the lambs (Table 3).

**Table 3:** Effect of different Green tea dust (GTD) supplementations (*n* = 6) on nutrient digestibility (%) after 84 days feeding trial.

Digestibility	Control	GTD-0.75	GTD-1.5	SEM	*P-value*
Dry matter	55.7	52.8	54.3	2.28	0.677
Organic matter	45.5	46.2	46.0	3.14	0.987
Protein	41.8	37.2	45.7	5.62	0.570

The same letters in the same column indicate a non-significant different at *P* > 0.05.

However, GTD-1.5 treatment resulted in decreased (*P* < 0.05) FEC in the lambs in comparison with the other treatments at day 42 and day 56 (Table 4).

**Table 4:** Effect of different green tea dust (GTD) supplementations (*n* = 6) on fecal egg counts (FEC, eggs/g feces) during 72 feeding trial.

Measurement	Control	GTD-0.75	GTD-1.5	SEM	*P-value*
FEC Day-0	773	827	827	91.2	0.893
FEC Day-14	1387	2167	1487	480	0.475
FEC Day-28	1927	2673	1247	622	0.298
FEC Day-42	1880^AB^	4553^A^	1207^B^	916	0.048
FEC Day-56	4480^A^	2167^AB^	1527^B^	803	0.048
FEC Day-72	3660	2933	1843	945	0.414

Different letters in the same column indicate a significant different at *P* ≤ 0.05.

## 4 Discussion

### 4.1 Performance

Ruano, Carolino and Mateus (2017) reported that Strongylus sp. infection in sheep can cause a significant decrease in growth and meat production. Meanwhile, Vlassoff, Leathwick and Heath (2001) revealed that Strongylus sp., a nematode parasite in sheep, has a complex life cycle involving development within the host and the environment. The larvae of Strongylus sp. hatch in the feces of the host and undergo several stages of development before becoming infective. Infected larvae can interfere intestinal function and absorption of the host's nutrients, which can lead to weight losses. The infection with Strongylus sp. in sheep resulted in inflammation of the digestive tract and disruption of the immune system (Ruano et al., 2017). This can interfere with animal digestion and metabolism, which in turn affects their growth and development (Ruano et al., 2017). GTD, which contains bioactive compounds such as tannins, has been shown to not only have anthelmintic properties but also to promote growth by improving nutrient utilization and reducing the negative impacts of parasitic infections. Tannins in GTD can bind to proteins, protecting them from degradation in the rumen and allowing for more efficient absorption in the small intestines. This improved protein availability can enhance growth rates and overall animal productivity. Additionally, by mitigating the effects of parasitic infections, GTD helps maintain intestinal health, leading to better feed efficiency and growth (Hoste, Jackson, Athanasiadou, Thamsborg & Hoskin, 2006; Marume, Chimonyo & Dzama, 2012; Vargas-Magaña et al., 2014). Notably, Ramdani et al. (2020) reported that the inclusion of 1.5% GTD in the concentrate of fattlening lambs increased daily gain of the lambs signifiantly without any harmful impacts on feed consumption and nutrient digestibility, underscoring its potential as a valuable feed additive.

### 4.2 Nutrient digestibility

A previous study conducted by Nasehi, Torbatinejad, Rezaie and Ghoorchi (2018) revealed that green tea waste containing bioactive compounds such as polyphenols and catechins had antioxidant and anti-inflammatory effects. These compounds can help reducing inflammation in the digestive tract caused by Strongylus sp. infection, thereby increasing the absorption of nutrients and health of the sheep (Nasehi et al., 2018). Ruminants fed with tannin-rich diets showed a decrease in rumen feed degradability and their digestibility (Frutos, Hervás, Giráldez & Mantecón, 2004). However, the current study showed that there was no significant difference in the nutrient digestibility of the lambs between the control and treatment groups.

Galicia-Aguilar et al. (2012) reported that a dietary tannin-rich additive decreased DM digestibility in sheep infected with gastrointestinal nematode because condensed tannins might have a negative nutritional effect (Waghorn, 2008). The anti-nutritional effect of condensed tannins is seen through a reduction in feed protein availability and a depression in digestive tract enzyme activities (Silanikove, Gilboa, Nir, Perevolotsky & Nitsan, 1996). Scharenberg et al. (2008) reported that feeding Sainfoin rich in protein and tannins increased the nitrogen retention and organic matter apparent digestibility but NDF and ADF digestibility were decreased.

### 4.3 Fecal egg counts

The number of intestinal helminths present in the feces is fluctuated and is closely associated with the life cycle of the parasites and determining the number of eggs produced. Several studies have explored this dynamic relationship between the worm's life cycle and egg productions, shedding light on the patterns of rise and fall in worm populations within the host's gastrointestinal tract. As elucidated by Ruano et al. (2017), the life cycle of intestinal worms consists of various developmental stages including egg, larva, and adult worm. The number of eggs produced by adult worms potentially increase worm population. When conditions are conducive, adult worms can lay a substantial number of eggs, contributing to a higher prevalence of eggs in the fecal matter. This scenario explains the observed increase in worm egg counts in the feces.

Conversely, fluctuations in worm populations can also be attributed to factors that influence the various stages of their life cycle. Dybing, Fleming and Adams (2013) stated that the availability of suitable hosts, environmental conditions, and host immunity can affect the survival and development of helminth larvae. Under unfavorable conditions or increased immune responses by the host, larval development may be hampered, resulting in reduced egg production by adult worms. This, in turn, leads to a decline in the quantity of worm eggs detected in the fecal matter. These fluctuations are closely aligned with the expected time frames for the completion of the worm's life cycle. Peaks in egg counts corresponds to periods when adult worms produce a large number of eggs, followed by declines as larval development is affected by factors such as seasonal changes and host immune responses.

Several studies have highlighted the anthelminthic potential of various plants rich in bioactive compounds (Ramdani et al., 2023). One of the above plants is tea leaves (*Camellia Sinensis*) due to its rich content of bioactive polyphenols in particular to green tea (Ramdani et al., 2022). Julaeha et al. (2021) reported that dietary *Jatropha multifida* rich in total phenols and tannins could increase the performance and reduce *Trichostrongylus* sp. Infection in growing lambs. The advantageous impact of polyphenols on combating gastrointestinal nematodes in ruminants can be indirectly by enhancing the host's response to parasites. The ability to bind to proteins by phenolic tannins can protect proteins from rumen degradation but increase protein flow and absorption of amino acids in the small intestine. Increased intestinal protein supply is known to increase host homeostasis and immune responses to worms (Julaeha et al., 2021). Phenolic tannins could bind to the free protein in the wells for larvae nutrition; reduced nutrient availability in the wells could have resulted in larvae starvation and death. Condensed tannins may also bind to the cuticle of larvae, which is high in glycoprotein. During the trans cuticular diffusion process, bioactive compounds such as alkaloids, flavonoids, saponins, and phenolic tannins move into the body of larvae so that the process of energy formation and absorption of nutrients is disrupted. In this condition, the larvae will die because of the reduced amount of energy and electrolytes inhibit larval development (Julaeha et al., 2021).

Dietary GTD helped growing lambs to not only increase the performance without affecting nutrient digestibility (Ramdani et al., 2020) but also helped *Strongyles* worms-infected lambs maintaining their body weights from a potential significant weight losses and reduced the nematode infection significantly especially after 42 days feeding trial as can be seen in this current study. In addition, improved performance and health of ruminants by decreasing nematode infections will increase the welfare status of the animals (Anugerah et al., 2023).

Green tea dust is by-product in green tea fabrications but it has comparable amounts of protein and phenolic tannins in comparison with the original green tea. Unfortunately, the particle size of GTD is very finely or dusty so that GTD is categorized as non-grade green tea resulting its price considerably cheaper than that the original green tea.

## 5 Conclusion

Dietary GTD helped *Strongyles* worms-infected lambs maintaining their body weights from a potential significant weight losses and reduced the nematode infection significantly after 42 days feeding trial. About 1.5% dietary GTD is suggested to maintain the productivity, health, and welfare statuses of rearing lambs.

## Ethics approval

Padjadjaran University Research Ethics Commission Number: 307/UN6.KEP/EC/2021.

## Declaration of generative AI and AI-assisted technologies in the writing process

The writing process, devoid of generative AI and AI-assisted technologies, relies solely on human creativity and manual cognitive efforts.

## Financial support statement

RKI 2023 research grant number: 2213/UN6.3.1/TU.00/2023.

## CRediT authorship contribution statement

**Diky. Ramdani:** Writing – review & editing, Validation, Supervision, Project administration, Methodology, Funding acquisition, Conceptualization. **Aldyansah Putra. Utama:** . **Ririn Siti. Rahmatillah:** Writing – original draft, Software, Formal analysis, Data curation. **Juju. Julaeha:** Investigation, Conceptualization. **Novi. Mayasari:** Supervision, Conceptualization. **Ken Ratu Gharizah. Alhuur:** Conceptualization. **Nanik. Hidayatik:** Writing – review & editing. **Anuraga. Jayanegara:** Writing – review & editing.

## Declaration of competing interest

The authors declare that they have no known competing financial interests or personal relationships that could have appeared to influence the work reported in this paper.

## Data Availability

The datasets generated and/or analyzed during the current study are not publicly opened, but are available from the corresponding author on a reasonable request.
